# Safety and Efficacy of a Novel Shunt Surgery Combined with Foam Sclerotherapy of Varices for Prehepatic Portal Hypertension: A Pilot Study

**DOI:** 10.6061/clinics/2019/e704

**Published:** 2019-08-13

**Authors:** Zhe Zhang, Xueming Chen, Chenyu Li, Hai Feng, Hongzhi Yu, Renming Zhu, Tianyou Wang

**Affiliations:** I Department of Vascular Surgery, Beijing Friendship Hospital, Capital Medical University, ChinaChina; II Department of Thoracic Surgery, Beijing Friendship Hospital, Capital Medical University, ChinaChina

**Keywords:** Prehepatic Portal Hypertension, Foam Sclerotherapy, Shunt Surgery, Gastroesophageal Varices

## Abstract

**OBJECTIVES:**

This pilot study investigated the safety and efficacy of a novel shunt surgery combined with foam sclerotherapy of varices in patients with prehepatic portal hypertension.

**METHODS:**

Twenty-seven patients who were diagnosed with prehepatic portal hypertension and underwent shunt surgeries were divided into three groups by surgery type: shunt surgery alone (Group A), shunt surgery and devascularization (Group B), and shunt surgery combined with foam sclerotherapy (Group C). Between-group differences in operation time, intraoperative blood loss, portal pressure decrease, postoperative complications, rebleeding rates, encephalopathy, mortality rates and remission of gastroesophageal varices were compared.

**RESULTS:**

Groups A, B and C had similar operation times, intraoperative bleeding, and portal pressure decrease. The remission rates of varices differed significantly (p<0.001): one patient in Group A and 6 patients in Group B had partial response, and all 9 patients in Group C had remission (2 complete, 7 partial). Two Group A patients and one Group B patient developed recurrent gastrointestinal bleeding postoperatively within 12 months. No postoperative recurrence or bleeding was observed in Group C, and no sclerotherapy-related complications were observed.

**CONCLUSIONS:**

Shunt surgery combined with foam sclerotherapy obliterates varices more effectively than shunt surgery alone does, decreasing the risk of postoperative rebleeding from residual gastroesophageal varices. This novel surgery is safe and effective with good short-term outcomes.

## INTRODUCTION

Prehepatic portal hypertension (PPH) is elevated pressure of the portal vein system as a result of extrahepatic portal venous obstruction (EHPVO) or presence of arterioportal fistula. Stenosis, obstruction, or thrombosis of the extrahepatic portal vein system may result in EHPVO 1, which is a major cause of noncirrhotic portal hypertension and variceal bleeding in children 2,3, as well as a common cause of upper gastrointestinal bleeding in adults 4.

The most severe and common complication of PPH is gastrointestinal bleeding. Approximately 5% to 30% of patients will die of gastrointestinal bleeding if timely treatment is not administered 5. Thus, the goal of treatment for PPH is to control and prevent gastrointestinal bleeding. Vasoactive drugs (somatostatin and octreotide) can prevent bleeding, and propranolol is used to reduce portal pressure 6, but the effects of these drugs are limited.

Current treatments for PPH mainly include endoscopic therapy, interventional therapy and surgery 5,7,8. Endoscopic sclerotherapy (EST) or band ligation are commonly used to treat gastroesophageal varices 7. However, although endoscopic treatment is less invasive, long-term results have been reported to be poorer than those of other methods 9. Endoscopic therapy does not effectively reduce portal pressure, and bleeding typically recurs 10. Among the interventional options for treating PPH, placement of a transjugular intrahepatic portosystemic shunt (TIPS) is the standard treatment for bleeding due to esophageal varices as a result of PPH that is refractory to endoscopic therapy 11.

Surgery remains an effective strategy for the control and prevention of PPH-related gastrointestinal bleeding 12 and is especially applicable to patients for whom endoscopic therapy has failed 13. Surgical interventions include various shunt surgeries and devascularizations, and the selection is determined by patients' individual conditions. Portosystemic shunts are the most common approach, including mesocaval, splenorenal and portacaval shunts 14. Devascularization, mainly including pericardial devascularization, esophageal transection and the Sugiura operation, directly blocks the paradoxical blood flow between the azygos vein and portal vein, precisely controlling the acute bleeding of ruptured esophageal varices and assuring blood flow to the liver 15. However, devascularization alone cannot reduce portal vein pressure and may result in deterioration of portal hypertensive gastropathy, causing thrombosis in the portal vein and increasing the risk of rebleeding 15. Shunt surgery and devascularization are also used together. For example, mesocaval shunts or splenorenal shunts can be combined with pericardial or gastric devascularization 8. Shunt surgery effectively reduces portal vein pressure, and devascularization removes the varicose veins, blocks blood circulation between the azygos vein and portal vein and maintains blood flow to the liver, preventing variceal rebleeding and encephalopathy.

Surgeons in our hospital are still exploring surgical approaches for PPH. Previously, we employed portosystemic shunts such as the mesocaval shunt and proximal splenorenal shunt. For the past five years, we have used shunt surgery combined with devascularization. In the last two years, we have used shunt surgery combined with foam sclerotherapy of the varices as an alternative method to treat PPH, although the safety and efficacy of this operation has not yet been reported. Therefore, this pilot study was undertaken to compare the safety and efficacy of our novel surgical approach with that of conventional surgeries in obliterating esophagogastric varices and preventing postoperative rebleeding.

## PATIENTS AND METHODS

### Patient population

In this retrospective, comparative pilot study, data from 33 patients who were diagnosed with PPH based on findings of abdominal ultrasonography and enhanced computer tomography (CT) between January 2007 and March 2016 were collected. The data of 4 patients who received conservative treatment were excluded from the analysis. Of the 29 patients who received surgical interventions, 1 received exploratory laparotomy, 1 underwent splenectomy alone, and 27 received shunt surgery or shunt surgery combined with other surgical interventions. Data from the 27 shunt surgery patients were included for analysis and divided into 3 groups according to the type of surgery received: Group A (n=10) included patients who received shunt surgery alone; Group B (n=8) included patients who received shunt surgery combined with devascularization; and Group C (n=9) included patients who received shunt surgery combined with foam sclerotherapy.

All included patients had portal obstruction due to any cause, resulting in increased blood flow and symptoms of portal hypertension. Ultrasonography and CT showed filling defects in the main portal vein, absence of a main portal vein or portal cavernoma. The protocol for this retrospective study was reviewed and approved by the Institutional Review Board of Beijing Friendship Hospital. Signed consent was not needed because the retrospective data were deidentified.

### Surgical procedures

All 27 patients in the three groups underwent surgery by the same team of surgeons. Foam sclerotherapy, for Group C patients only, was administered during the procedure by the surgeon after performing the shunt surgery.

#### Mesocaval shunt surgery

Patients received general anesthesia, and a midline incision was made for laparotomy. Portal pressure was measured using the piezometer before shunting. The major superior mesenteric vein and its branches were exposed after the mesenteric tissues were separated and ligated. Then, the inferior vena cava was exposed through the posterior peritoneum. The superior mesenteric vein and inferior vena cava were clamped, and 10-15 mm incisions were made on the vessel wall. Then, 5-0 Prolene suture was used for side-to-side anastomosis. If a side-to-side suture was impossible due to the long distance between the superior mesenteric vein and the inferior vena cava, an artificial (prosthetic) blood vessel with a diameter of 8-12 mm (W.L. Gore & Associates, Newark, DE, USA) was used to anastomose. After shunting, the pressure of the omentum vein was measured again and recorded, followed by wound closure.

#### Splenorenal shunt surgery

After general anesthesia, a midline incision or an “L” type incision was made. Before exploration, portal vein pressure was measured, followed by splenectomy. Approximately 3-4 cm of the splenic vein was reserved for further anastomosis, and the renal vein was exposed through the posterior peritoneum. After an incision was made on the renal vein, an end-to-side anastomosis of the splenic and renal veins was established. A drainage tube was placed in the splenic fossa, and the portal vein pressure was measured again before wound closure.

#### Shunt surgery combined with devascularization

After shunt surgery, the gastric fundus was exposed, and the proximal branches of gastroepiploic vessels and short gastric veins were exposed along the greater and lesser curvature, followed by ligation. Some varicose veins at the gastric body or fundus were sutured through the gastric wall.

#### Shunt surgery combined with foam sclerotherapy

**Figure 1:**
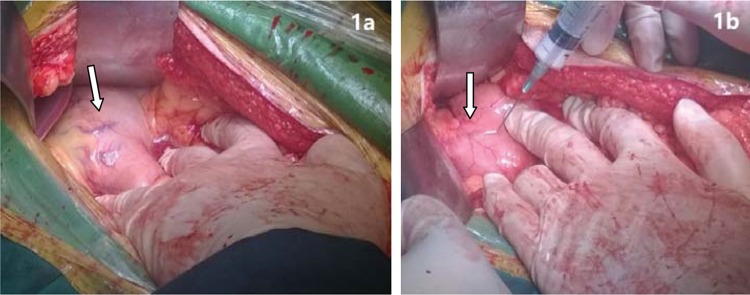
Varicose veins before and after treatment. **a)** Varicose veins at lesser gastric curvature. **b)** Occlusion of varicose veins after injection of polidocanol foam.

**Figure 2:**
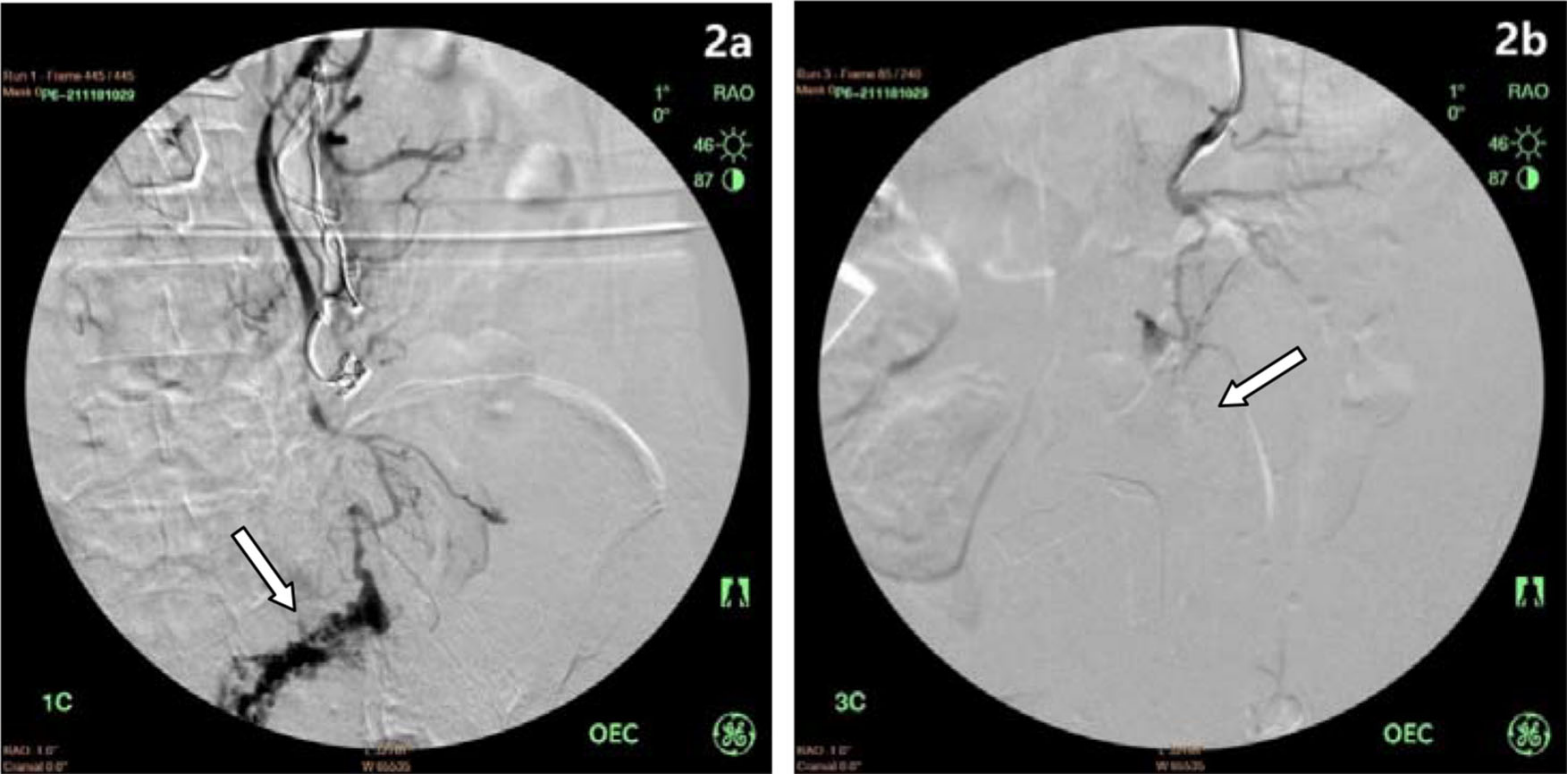
Rectal varices before and after treatment. **a)** Rectal varices on intraoperative angiography of the inferior mesenteric vein. **b)** Occlusion of rectal varicose veins after injection of polidocanol foam.

After shunt surgery, the gastric varicose veins were exposed. Foam sclerosant was made up of 2 ml of 1% polidocanol (Aethoxysklerol, Kreussler, Wiesbaden, Germany) mixed with 8 ml of air, as previously described by Tessari et al. 16. Then, 2-5 ml of foam was injected into the major varicose vein. After injection, the puncture site vessel was ligated, followed by injection of the foam into other varicose veins (Figure 1a, 1b). Foam sclerosant at different concentrations was selected based on the size of the varicose veins; generally, 1% foam sclerosant was used when the vessel was smaller than 6 mm in diameter. However, if the varices exceeded 6 mm in diameter, 3% polidocanol was applied. In one patient with rectal varices, the inferior mesenteric vein was exposed, and a 4 Fr sheath was inserted, followed by a guide wire (0.035 inch) and a 4 Fr catheter inserted into the distal inferior mesenteric vein. Contrast agent was injected to visualize the distal inferior mesenteric vein and rectal varices. After withdrawal of the guide wire, 3% polidocanol foam (10 ml) was injected to occlude the rectal varices. A second angiography showed the absence of varicose veins (Figure 2a, 2b).

### Definitions and measures

The complete remission of gastroesophageal varices was defined as an absence of any varicose vein in the esophagus and gastric fundus on CT and/or gastric endoscopy; partial remission was defined as the presence of but a reduction in varicose veins in the esophagus and gastric fundus; no-remission was defined as unchanged varicose veins in the esophagus and gastric fundus. The remission rate was calculated as the proportion of patients with partial remission or complete remission to total patients.

### Intraoperative and postoperative observations and follow-up

Operation time, intraoperative blood loss, reduction in portal pressure after shunt surgery (difference between portal pressure before and portal pressure after shunt surgery), incidence of postoperative complications, and mortality within 30 days after surgery were compared between groups. All patients were examined for patency of shunts before discharge. All patients had ammonia determination before being discharged and after receiving surgery; during the postoperative follow-up after discharge, patients without symptoms of encephalopathy did not have ammonia determinations at that time.

Patients were followed for 12 months after surgery, and the incidence of rebleeding (hematemesis or bloody stool due to variceal bleeding), the incidence of hepatic encephalopathy and the survival rates were compared among groups at 12 months. Findings from abdominal CT or gastric endoscopy before surgery and within 12 months after surgery were also compared. The remission rates of esophageal and gastric varices were compared after surgery among the three groups. The possible causes of rebleeding were explored, including the etiology (portal cavernoma or portal thrombosis), reduction in portal pressure after shunt surgery and remission of gastroesophageal varices. Associations between these factors and rebleeding were further evaluated.

### Statistical analysis

Patient characteristics are presented as n (%) for categorical data and mean with range (min. to max.) for continuous data. The characteristics during surgery, within 30 days and within one year of surgery are presented as the mean ± standard deviation (SD) for operation time, blood loss and reduction in portal pressure, and n (%) for other categorical data of safety and efficacy evaluations. Differences among groups were compared using the Kruskal-Wallis test for continuous data and Fisher's exact test for categorical data. The Mann-Whitney U test was also applied for continuous data between two groups. All statistical analyses were carried out using IBM SPSS statistical software version 22 for Windows (IBM Corp., Armonk, NY, USA).

## RESULTS

### Patient characteristics

In this retrospective pilot study, the data of 27 patients with PPH (15 males and 12 females) with a mean age of 35.5 years (range: 8 to 62 years) were collected from January 2007 to March 2016. All patients were diagnosed via enhanced abdominal CT screening, including 16 patients with portal cavernoma and 11 with chronic portal venous thrombosis. Comorbidities included 27 patients with gastroesophageal varices, 20 with splenomegaly, 14 with hypersplenism, 11 with liver cirrhosis, 10 with ascites and one with rectal varices. The medical histories revealed that 25 patients had gastrointestinal hemorrhage, and 2 had portal hypertensive gastropathy. Eighteen patients had Child-Pugh class A cirrhosis, and 9 had class B (Table 1).

### Safety and efficacy

In Group A, a mesocaval shunt was placed in 5 patients, a mesocaval shunt using an artificial vessel in 2 patients, a splenectomy + splenorenal shunt in 2 patients, and a splenocaval shunt using an artificial vessel in 1 patient. The mean operation time was 241.0±92.0 minutes, and the mean blood loss was 925.0±962.4 ml. The mean postoperative reduction in portal pressure was 10.9±6.7 cm H_2_O. After splenectomy, thrombocytosis was noted in one patient who was treated with anti-platelet therapy; one patient developed pleural and peritoneal effusion, which was alleviated after supplementation with albumin and diuretic treatment.

In Group B, mesocaval shunt + devascularization was performed in 6 patients, splenectomy + mesocaval shunt + devascularization in 1 patient, and splenectomy + splenorenal shunt + devascularization in 1 patient. The mean operation time was 246.2±31.7 minutes, and the mean intraoperative blood loss was 475.0±315.1 ml. The mean postoperative reduction in portal pressure was 9.2±4.6 cm H_2_O. After splenectomy, one patient developed a splenic fossa abscess and an abdominal infection, which was corrected by splenic fossa drainage; one patient developed abdominal bleeding within 5 hours after surgery, so a second exploratory laparotomy was performed, and the patients had a smooth recovery.

In Group C, mesocaval shunt + foam sclerotherapy was performed in 8 patients, and a mesocaval shunt was placed using an artificial vessel + foam sclerotherapy in 1 patient. The median volume of polidocanol used in surgery was 16.9 ml. The mean operation time was 242.2±55.7 minutes, and the mean intraoperative blood loss was 511.1±493.6 ml. The mean reduction in portal pressure was 9.8±3.4 cm H_2_O. One patient developed upper gastrointestinal bleeding within 3 days after surgery, and endoscopy showed ulcer-related mucosal bleeding due to the shedding of tissue glue that was administered before surgery; this patient recovered after blood transfusion and anti-acid treatment. One patient developed persistent nasal bleeding due to thrombocytopenia, which resolved after focal hemostasis and plasma transfusion. Sclerotherapy-related complications were not observed in this group.

In Group A, 2 patients developed recurrent gastrointestinal bleeding at 4 and 10 months after surgery, of whom 1 received endoscopic hemostasis and the other received conservative therapy. One patient developed hepatic encephalopathy at 5 months after surgery but recovered after corresponding treatment, and similar symptoms were not observed again. In Group B, recurrent bloody stool was observed in 1 patient at 10.5 months after surgery but resolved after conservative therapy. In the follow-up period, obstruction of the shunt was observed in 1 patient, but without rebleeding, and no patients developed hepatic encephalopathy. In Group C, no postoperative rebleeding was observed; 1 patient developed hepatic encephalopathy at 2 months after surgery, which resolved after corresponding treatment and was not observed thereafter.

**Figure 3:**
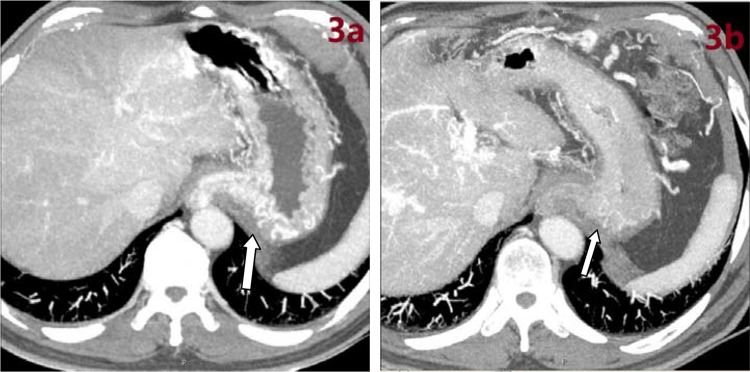
Perioperative enhanced CT images. **a)** Gastroesophageal varices on preoperative enhanced CT. **b)** Absence of varicose veins on postoperative enhanced CT after foam sclerotherapy in the same patient.

**Figure 4:**
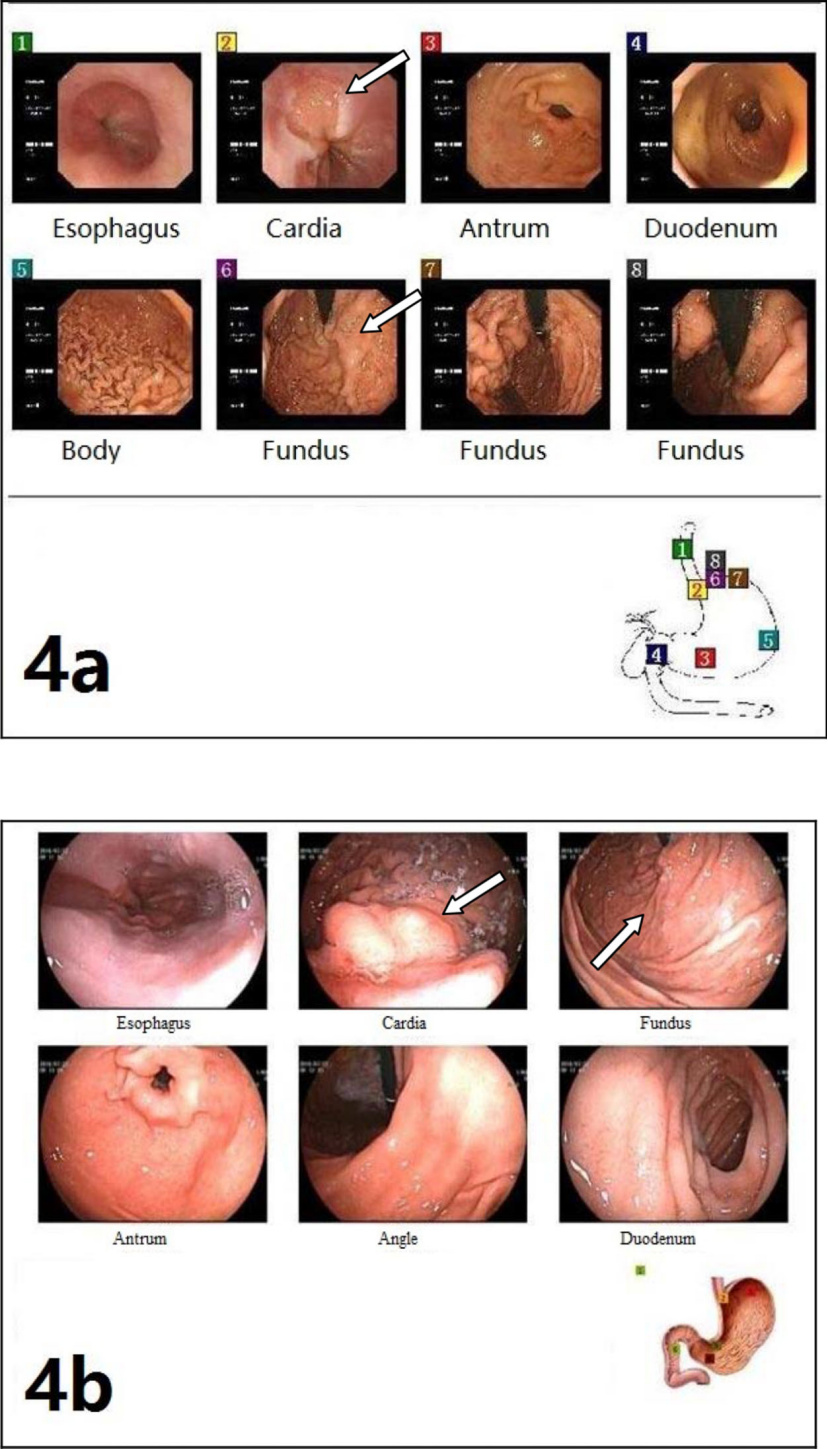
Endoscopy images. **a)** Preoperative endoscopy showing gastroesophageal varices. **b)** Endoscopy review of the same patient showing obviously decreased gastric varices but residual esophageal varices postoperatively.

Table 2 presents the operative parameters by group. During the operations, all three groups had similar operation times, intraoperative blood loss, and a reduction in portal pressure (all *p*-values>0.05) (Table 2). No patients in any group died within 30 days after surgery. A total of 6 patients, including 2 in Group A, 2 in Group B, and 2 in Group C, developed complications within 30 days after surgery. Within 12 months after surgery, a total of 3 patients had recurrent bleeding at 4 months, 10 months, and 10.5 months; 2 patients had encephalopathy, and one experienced shunt occlusion. The remission rates of varices were significantly different between the three groups (*p*<0.001): one patient in Group A and 6 patients in Group B had partial remission; 9 patients in Group C had remission, including 2 with complete remission (Figure 3a, 3b) and 7 with partial remission (Figure 4a, 4b) (1 had complete remission of rectal varices). No patients in any group died within 12 months after surgery (Table 2).

Table 3 presents associations between postoperative bleeding and other factors. No associations were found between the included factors (all *p*-value>0.05) (Table 3).

## DISCUSSION

The results of this retrospective pilot study showed that the safety and efficacy of shunt surgery combined with foam sclerotherapy were comparable to the safety and efficacy of shunt surgery plus devascularization for patients with PPH. Notably, both shunt surgery plus foam sclerotherapy and shunt surgery plus devascularization were superior to shunt surgery alone in terms of preventing recurrence and rebleeding.

The goal of PPH treatment is the control and prevention of gastrointestinal bleeding. Unlike in patients with hepatic cirrhosis-related portal hypertension, gastroesophageal varices in PPH are evidently causing more blood loss, and concomitant esophageal and gastric varices are more common (31%-44% *vs*. 22%). Some PPH patients have concomitant rectal and anal varices. Isolated varicose veins are found in approximately 6% of PPH patients, and ectopic or duodenal varices are also common 17,18.

Surgery is the conventional treatment of choice for PPH according to the guidelines of the American Association for the Study of Liver Diseases 19, but with the development of minimally invasive treatment, endoscopy and interventional procedures have become the preferred treatments. Evidence shows that the efficacy of hemostasis by endoscopy is as high as 90% in the emergency department 4, and endoscopy is especially applicable in patients with acute bleeding who are not candidates for open surgery. Endoscopy is also employed to prevent bleeding, but it cannot reduce portal pressure and may increase the risk for recurrent gastric varices and ectopic varices 20. Bleeding recurrence is common after endoscopy, with a rate as high as 40%-70% 21,22. Therefore, regular follow-up and repeated treatments are needed for patients who receive endoscopy alone, but this therapeutic pattern is often inapplicable in developing countries. Interventional procedures such as TIPS and percutaneous mesocaval shunt creation are minimally invasive and suitable for patients who cannot undergo open surgery, although portal vein obstruction or chronic portal vein thrombosis remains a relative contraindication to TIPS 11,23. Additionally, the technique for percutaneous mesocaval shunt placement is a challenging and potentially precarious procedural approach requiring transmesenteric, intraperitoneal vessel puncture and clinical experience is limited. PPH may also cause other complications, such as splenomegaly, hypersplenism, growth and development disorders, portal hypertensive gastropathy and ectopic varices, which cannot be managed by endoscopy. Thus, treatment must be individualized, and comprehensive treatment is required for PPH patients.

To date, surgery remains an effective treatment for PPH, and the long-term survival rate of PPH is higher than 95% after shunt surgery 24. Its morbidity and mortality can be minimized by experienced surgeons. However, few studies have compared the efficacy between shunt surgery and endoscopy. A single-center, randomized, controlled study indicated that the mortality was comparable between shunt surgery and endoscopy in PPH patients, but the rate of bleeding recurrence in the endoscopy group (22.6%) was significantly higher than that in the shunt surgery group (3.3%), and a high rate of treatment failure was also found in the endoscopy group (19.4% vs. 6.7%) 25. Surgery is also reported to improve pediatric patients' growth and development, attenuate the development of varices, and relieve PPH-related biliary diseases 26,27. Accepted indications for surgery for PPH include 1 acute gastrointestinal bleeding that is nonresponsive to endoscopic hemostasis; 2 gastric and ectopic varicose veins for which endoscopic treatment is too difficult to perform; 3 the presence of splenomegaly, hypersplenism, growth and development disorders, portal hypertensive biliary tract disease or other diseases; and 4 when patients have difficulty making repeat hospital visits and are willing to receive one-time treatment 12.

In the present study, all patients had portal obstruction, so the mesocaval shunt technique was employed. The blood flow shunted is less than that with the portocaval shunt, reducing the risk for postoperative hepatic encephalopathy. Because the long distance between the superior mesenteric vein and inferior vena cava may make direct anastomosis difficult, an artificial (prosthetic) vessel can be used for the shunt between these vessels. However, artificial vessels have a low long-term patency rate and cannot grow over time; thus, the use of a prosthetic is inapplicable in children. For patients with severe splenomegaly or hypersplenism, shunt surgery should be prepared during splenectomy, and a proximal splenorenal shunt is preferred for surgical intervention. Splenectomy cannot effectively reduce the portal pressure or relieve esophageal and gastric varicosities but can increase the susceptibility to splenic vein thrombosis, which may risk involvement of the portal vein and superior mesenteric vein. This condition requires performing shunt surgery 28, and splenectomy alone should be avoided for these patients.

Although shunt surgery effectively reduces portal pressure, recurrent bleeding is still possible after surgery due to obstruction of shunt vessels and/or residual varicose veins 29. PPH patients may have large varicose veins, and even though pressure is reduced, the dilated vessels cannot remodel, and thin-walled veins may rupture. In the present study, although the absence of varices was not associated with postoperative recurrence of bleeding, three patients with recurrent bleeding had no improvement in varices after surgery, indicating that residual varicose veins may increase the risk for bleeding recurrence. Therefore, in shunt surgery, eliminating varices is necessary for concomitant devascularization or concomitant foam sclerotherapy.

Theoretically, the combined use of shunt and devascularization not only reduces portal pressure but also removes residual varicose veins, helping to prevent recurrent bleeding. Feng et al. 30 achieved favorable efficacy using mesocaval artificial vessel shunts, ligation of gastric fundal veins and coronary veins of the stomach, and partial splenectomy. In that study of 100 patients, no recurrence, bleeding or hepatic encephalopathy were observed during follow-up. In the present study, among the eight patients (Group B) who underwent surgical intervention with shunt creation and devascularization and received a postoperative CT scan, six patients had remission of varices, indicating that the combined use of a shunt and devascularization is effective for attenuating varices. However, two patients still had no attenuation of varices, which might be ascribed to residual varicose veins due to incomplete devascularization. In addition, devascularization sometimes requires expansion of the surgical field to expose the varicose veins completely, which may increase surgical risk and difficulty compared to shunt surgery alone. Therefore, we attempted to combine a shunt with foam sclerotherapy to replace conventional shunt surgery, aiming to effectively remove the varicose veins and simplify the surgery.

Foam sclerotherapy has been widely used in treating limb varices, endoscopic sclerotherapy and balloon-occluded retrograde transvenous obliteration (BRTO), and can effectively close varicose veins. However, direct injection of sclerosant into fundal varicose veins during open surgery has not been reported. Based on the classification of gastric varices, some patients have both esophageal and gastric varices with connections between the varicose veins (GOV1 type and GOV2 type) 31. After injecting sclerosant into fundal varicose veins, it may be distributed to the entire varices system via communicating collaterals and even further up to the esophageal veins. One-time injection may cause the obstruction of a wide range varices, which increases the efficacy of varicose vein removal and decreases surgical trauma and difficulty. In addition, ectopic varicose veins (e.g., rectal veins) that are difficult to expose anatomically can also be managed with hybrid surgery. The catheter can be inserted into the distal lesion with the aid of digital subtraction angiography (DSA), and the sclerosant is then injected to obstruct the varicose veins, which may also simplify this complex surgery.

In the present study, we used polidocanol, a liquid embolizing agent of the sclerosing class, as the foam sclerotic reagent and found it to be safe and effective. This reagent has been widely used in the treatment of limb varicosis and venous malformation, as well as in endoscopic treatment 32,33. Polidocanol is mixed with gas (1:4) to form the foam sclerotic reagent. This reagent can damage vascular endothelial cells chemically, leading to thrombosis, fibrosis and final permanent occlusion. Foam sclerosant has several advantages over liquid sclerosant: a lower dose of foam sclerosant can be used, increasing safety, and the distribution of foam sclerosant is even greater in the vessels, increasing the area of sclerosant contacting vessel walls and elevating the efficacy of sclerotherapy 34. Darke et al. 35 reported that polidocanol foam effectively induced occlusion of varicose veins of the lower limbs with >90% overall effectiveness. The results of a multicenter study showed that the efficacy of foam sclerotherapy (69%) was significantly higher than that of liquid sclerotherapy (27%) in patients with lower extremity varices 36. Foam sclerosant is also used in BRTO. Several studies employed retrograde injection of polidocanol foam via a spontaneous gastrorenal shunt to obstruct fundal varicose veins, resulting in an obstruction rate as high as 91%-100%, and only one patient had bleeding recurrence during follow up 37-39.

Foam sclerotherapy is usually performed after shunt surgery because the shunt reduces the pressure of the esophageal and fundal veins and decreases the blood flow from the varicose veins into systemic circulation. Therefore, injection of foam sclerosant after shunt surgery may not induce systemic embolism. In shunt surgery, the varicose veins at the greater curvature, lesser curvature and fundus are completely exposed, and 2-5 ml of polidocanol foam is then injected into the target vessels. Before injection, withdrawal of the syringe is needed to ensure that the needle localizes in the vessel lumen. Stable injection may be difficult when the target vessels are small or curled or if the space is narrow. In such cases, an intravenous infusion needle with extended tubes can be used to inject the foam sclerosant, increasing needle stability. The sclerosant dose should be determined based on the severity of varices before and during surgery. The sclerosant dose can usually reference that used in the treatment of limb varices, in which 2-5 ml of sclerosant is injected into each vessel, and the dose can be increased for larger varicose veins. Polidocanol is available in concentrations of 0.5%, 1% and 3%; 1% polidocanol is most commonly used in treating fundal varices, while 3% polidocanol is used if the diameter of varicose veins is larger than 6 mm. For fundal varices, pressurization is inapplicable after injection of foam sclerosant, and unsuccessful obstruction of the target vessels may increase the possibility of recirculation. Studies with long follow-up are needed to determine whether this will affect long-term efficacy.

In the present study, although none of the patients in Group C who received shunt + foam sclerotherapy had rebleeding, 20% of patients receiving shunt surgery alone (Group A) and 12.5% of patients receiving shunt surgery + devascularization (Group B) developed rebleeding within 12 months after surgery. For short-term efficacy, shunt surgery combined with foam sclerotherapy prevented postoperative recurrent bleeding more effectively, although significant differences were not observed among the three groups, so we cannot conclude that the new approach is superior to the conventional one. Further comparisons of findings from abdominal enhanced CT or gastroscopy before and after surgery showed decreased varices in more patients in Groups B and C than in Group A due to the treatment of fundal varicose veins in addition to shunt surgery in Groups B and C. Postoperative imaging in Group C showed that the esophageal and gastric varices decreased in all patients within 12 months, with complete remission in two patients and partial remission in seven patients, suggesting that obstruction of varicose veins with foam sclerosant has favorable efficacy. Notably, complete remission was not achieved in seven patients due to residual esophageal varices, which might be ascribed to the large vascular volume and the insufficient amount of sclerosant applied. Injection of foam sclerosant into the fundal veins alone may not spread into esophageal veins, but a decrease in fundal varices theoretically improves the prognosis of PPH patients. When the pressure gradient between the portal and systemic vein is <12 mmHg after shunt or TIPS placement, recurrent bleeding due to esophageal varices is rare, but fundal varices may still cause repeated bleeding 40. In addition, residual esophageal varices can also be managed by endoscopy, but the efficacy of endoscopy for fundal varices is inferior to that for esophageal varices 41. Therefore, a decrease in fundal varices after foam sclerotherapy is crucial for the control of postoperative recurrent bleeding.

Foam sclerotherapy is simpler to perform than devascularization. Although no significant differences were found in operation time or intraoperative blood loss in our patients who received foam sclerotherapy, this technique only requires exposure of fundal varicose veins and subsequent injection of sclerosant without excessive exposure of the surgical field. The hybrid surgery we used employs both interventional procedures and foam sclerotherapy, which can resolve difficulties common in conventional surgery (such as ectopic or rectal varices) with minimal invasion. In rectal and anal varices, redistribution of the portal vein may cause increased pressure in the inferior mesenteric vein. Although the rate of relevant bleeding is low (0.5%-10%), severe or even life-threatening consequences may still develop 42,43. One patient in Group C who had concomitant rectal varices had a history of repeated bloody stool and severe anemia before surgery. This lesion was hard to treat by conventional surgery, and rectal resection might be needed. In the shunt surgery, hybrid surgery was employed and involved catheter insertion via the inferior mesenteric vein and injection of sclerosant to obstruct the rectal varices. After the hybrid procedure, the rectal varices were occluded, and the bloody stool was relieved.

Typically, PPH patients have normal liver function or mild liver dysfunction, and the prognosis is good after surgery. In the present study, all patients had Child-Pugh class A or B cirrhosis and were tolerant of surgery with low surgical risk compared to those with decompensated liver function. In addition, no significant differences were found in 30-day postoperative mortality, operation times or intraoperative blood loss among the three groups, indicating similar safety between the different procedures. In one patient in Group C who developed upper gastrointestinal bleeding, endoscopy showed ulcer formation rather than ruptured varices after endoscopic glue obturation. Other studies have reported severe complications due to the shedding of tissue glue after endoscopic therapy 44,45. During the 12-month follow-up, all patients in the present study survived. Although hepatic encephalopathy was noted in one patient in Group A and one patient in Group C, it did not progress to hepatic coma and resolved after conservative treatment.

Compared with other sclerosants, such as sodium tetradecyl sulfate, polidocanol foam is reported to be more stable and has fewer side effects 45. The manufacturer's instructions advise a maximal safe dose of 2 mg/kg/d, suggesting that polidocanol at <2 mg/kg/d is safe. Evaluation of the safety and efficacy of polidocanol in 16804 patients who received sclerotherapy showed no allergy or death, indicating favorable safety 47. However, excessive amounts of polidocanol has been administered without sclerotherapy-related complications 48,49. Studies also indicate that foam sclerosant in the blood may not cause biological reactions, except for a transient increase in D-dimer 50. Although complications such as cardiac toxicity and pulmonary embolism do arise, the incidence is low 51, and lower doses of sclerosant are better than giving the maximal dose. In the present study, nine patients received 8-25 ml of 1% polidocanol (median: 16.9 ml). Given a liquid to air ratio of 1:4, the actual dose of polidocanol used was 16-50 mg. Rectal varices were treated in one patient with 10 ml of 3% polidocanol, which was equivalent to 60 mg of polidocanol and was lower than the maximal dose, and no sclerosant-related complications were observed, corroborating the safety of treating varices with polidocanol foam sclerosant.

### Limitations

The present pilot study still has several limitations. The incidence of PPH was low, leading to a small sample size. Because conducting a single-center, prospective, randomized controlled study of a new approach is difficult, the new approach can only be compared retrospectively to the conventional one. Additionally, only short-term efficacy was evaluated, and it is not clear whether risk exists for long-term vascular recanalization after foam sclerotherapy and whether significant differences may be found in long-term recurrent bleeding and survival rates among the surgical groups. Although no significant differences were found in patients' baseline characteristics (etiology, gender, age, complications and liver function), study bias may still exist, emphasizing the need for more multicenter, prospective, randomized controlled studies to confirm the results of the current trial.

## CONCLUSION

Shunt surgery combined with foam sclerotherapy has similar safety and efficacy as shunt surgery with devascularization. This combined procedure provides more effective variceal obliteration than shunt surgery alone does, and the combined procedure may reduce the risk of postoperative recurrent bleeding caused by residual gastroesophageal varices. In addition, foam sclerotherapy is easy to perform, is safe and has favorable short-term efficacy in combination with shunt surgery, but its long-term efficacy requires further study.

## AUTHOR CONTRIBUTIONS

Zhang Z was responsible for the study design, manuscript preparation, clinical studies and data analysis. Chen X: was responsible for the study concepts, study design, manuscript editing and clinical studies. Li C, Feng H, Yu H, Zhu R were responsible for the clinical studies and data acquisition. Wang T is the guarantor of integrity of the entire study and was responsible for the manuscript review.

## Figures and Tables

**Table 1:** Patient demographics and clinical characteristics (n=27).

	Total (n=27)	Group A (n=10)	Group B (n=8)	Group C (n=9)	*p*-value
Years					n/a
2007	1	1	0	0	
2008	3	3	0	0	
2009	2	2	0	0	
2010	4	4	0	0	
2012	1	0	1	0	
2013	2	0	2	0	
2014	3	0	3	0	
2015	8	0	2	6	
2016	3	0	0	3	
Sex					0.560
Males	15 (55.6)	6 (60)	3 (37.5)	6 (66.7)	
Females	12 (44.4)	4 (40)	5 (62.5)	3 (33.3)	
Age, years	35.5 (8-62)	29.9 (11-62)	29.4 (8-60)	47.2 (18-61)	0.108
Etiology					1.000
Portal cavernoma	16 (59.3)	6 (60)	5 (62.5)	5 (55.6)	
Chronic portal vein thrombosis	11 (40.7)	4 (40)	3 (37.5)	4 (44.4)	
Comorbidity					
Gastroesophageal varices	27 (100)	10 (100)	8 (100)	9 (100)	n/a
Splenomegaly	20 (74.1)	7 (70)	7 (87.5)	6 (66.7)	0.642
Hypersplenism	14 (51.9)	5 (50)	5 (62.5)	4 (44.4)	0.794
Liver cirrhosis	11 (40.7)	4 (40)	3 (37.5)	4 (44.4)	1.000
Ascites	10 (37)	3 (20)	5 (62.5)	2 (22.2)	0.247
Rectal varices	1 (3.7)	0 (0)	0 (0)	1 (11.1)	0.631
Medical history					0.512
Gastrointestinal hemorrhage	25 (92.6)	10 (100)	7 (87.5)	8 (88.9)	
Portal hypertensive gastropathy	2 (7.4)	0 (0)	1 (12.5)	1 (11.1)	
Child-Pugh class					0.098
A	18 (66.7)	7 (70)	3 (37.5)	8 (88.9)	
B	9 (33.3)	3 (30)	5 (62.5)	1 (11.1)	

Data are summarized as the number of patients by group for the years of patient enrollment; n (%) is shown for other categorical data; mean (range: min.-max.) is shown for age.

n/a, not available.

**Table 2:** Comparisons of intraoperative and postoperative characteristics (n=27).

	Total (n=27)	Group A (n=10)	Group B (n=8)	Group C (n=9)	*p*-value
Characteristics during surgery					
Operation time (min)	243.0±64.5	241.0±92.0	246.2±31.7	242.2±55.7	0.986
Intraoperative blood loss (ml)	653.7±683.7	925.0±962.4	475.0±315.1	511.1±493.6	0.613
Preshunting portal pressure level (cm H_2_O)	42.4±6.8	46.1±6.7	40±7.9	40.3±4.1	0.085
Postshunting portal pressure level (cm H_2_O)	32.3±5.3	35.2±5.3	30.7±5.0	30.5±4.8	0.099
Reduction level of portal pressure (cm H_2_O)	10.1±5.0	10.9±6.7	9.2±4.6	9.8±3.4	0.931
Within 30 days after surgery					
Complications	6 (22.2)	2 (20)	2 (25)	2 (22.2)	1.000
30-days mortality	0 (0)	0 (0)	0 (0)	0 (0)	NA
Within 12 months after surgery					
Recurrent bleeding	3 (11.1)	2 (20)	1 (12.5)	0 (0)	0.614
Encephalopathy	2 (7.4)	1 (10)	0 (0)	1 (11)	NA
Shunt occlusion	1 (3.7)	1 (10)	0 (0)	0 (0)	NA
Remission level of varices					<0.001*
Completed remission	2 (7.4)	0 (0)	0 (0)	2 (22.2)	
Partial remission	14 (51.9)	1 (10)	6 (75)	7 (77.8)	
No remission	11 (40.7)	9 (90)	2 (25)	0 (0)	
Remission rate	16 (59.3)	1 (10)	6 (75)	9 (100)	<0.001*
Mortality rate	0 (0)	0 (0)	0 (0)	0 (0)	NA

Data are summarized as the mean±SD for continuous data and as n(%) for categorical data. Differences among groups were compared using the Kruskal-Wallis test for continuous data and Fisher’s exact test for categorical data.

* *p*<0.05, indicates significant differences between groups.

NA, not available.

Three patients exhibited recurrent bleeding at 4 months, 10 months, and 10.5 months after the operation.

**Table 3:** Associations between postoperative bleeding and clinical factors.

	With postoperative bleeding (n=3^a^)	Without postoperative bleeding (n=24)	*p*-value
Operation time (min)	240±80	243.3±64.4	0.856
Intraoperative blood loss (ml)	750±1082.8	641.7±652.0	0.532
Reduction level of portal pressure (cm H_2_O)	7.7±0.6	10.4±5.3	0.532
Etiology			
Portal cavernoma	2 (66.7)	14 (58.3)	1.000
Chronic portal vein thrombosis	1 (33.3)	10 (41.7)	1.000
Encephalopathy	1 (33.3)	1 (4.2)	0.214
Complications	1 (33.3)	5 (20.8)	0.545
Remission rate within 12 months after operation	0 (0)	16 (66.7)	0.056

a Two patients in Group A and one patient in Group B had rebleeding within 12 months after operation.

Data are presented as n(%) and were analyzed using Mann-Whitney test for continuous data or Fishers' exact test for categorical data.

No significant associations were derived.
